# Pneumomastia following subdermal upper arm helium contouring treatment

**DOI:** 10.4102/sajr.v28i1.2899

**Published:** 2024-07-24

**Authors:** Joel H. Bortz, Joseph Lebovic

**Affiliations:** 1 LSG Imaging, Los Angeles, United States of America

**Keywords:** mammogram, subcutaneous emphysema, J-Plasma, Renuvion device, fascial planes

## Abstract

A case is presented of extensive pneumomastia seen on a screening mammogram of an asymptomatic patient who had helium plasma treatment 2 weeks earlier for flabby upper arms.

**Contribution:**

Rare complications of subcutaneous emphysema, following helium plasma treatment, have been discussed to highlight that such emphysema is usually self-limiting.

## Introduction

Gas in the breast (pneumomastia), after interventional breast procedures, is a common occurrence.^1,2^ Subcutaneous emphysema is also a cause of pneumomastia if there is no clinical history of interventional procedures, such as a recent breast biopsy.^1^ Pneumomastia, following abdominal laparoscopic surgery, occurs because of air tracking subcutaneously to the breast.^1,2^ Placement of a peripheral intravenous line in the arm of a patient was reported as the most likely cause of pneumomastia in the right breast.^3^ Surgical (subcutaneous) emphysema occurs following a puncture type injury to the chest wall, sinus cavity and bowel.^4^ It may also occur following infection with gas-forming micro-organisms.^4^ Air or gas enters the subcutaneous tissue and, if trapped, it stays in one place. It may also spread along connective tissue such as muscles and ligaments.^5^ Subcutaneous emphysema, following a combination of abdominal liposuction and J-Plasma, was recently reported in a patient who had bilateral thigh liposuction and then developed surgical emphysema in the chest and abdominal wall.^4^ Surgical emphysema under the right diaphragm was visualised in a patient, following Renuvion (J-Plasma) abdominal liposuction and was reported as a rare complication.^6^ Although a bronchopleural fistula is a rare risk post-thoracotomy, it may cause pneumomastia.^7^

This report describes a case of gas visualised on a screening mammogram of a patient who underwent treatment for flabby arms 2 weeks earlier. To the best of our knowledge, there are no cases in the literature of gas visualised in the breasts following this novel treatment.

## Ethical considerations

This article followed all ethical standards for research. Written consent was obtained from the patient.

## Patient presentation

**FIGURE 1:**
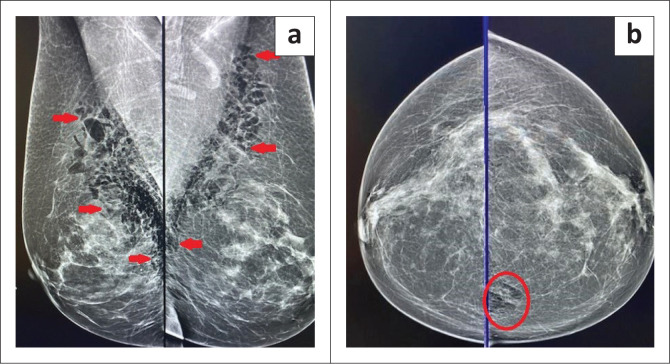
(a) Medio-lateral oblique views showing a large collection of gas (red arrows) in both breasts. (b) Cranio-caudal views showing a small amount of gas (red circle) posteriorly in the left breast.

A 73-year-old woman, with an unremarkable history, presented for her annual screening mammogram. The examination of both breasts was performed using a Siemens Mammomat Revelation system together with 3D tomosynthesis. Her mammogram images are presented in Figure 1a and b. There is a large collection of gas in the anterior and posterior aspects of both breasts as shown in the medio-lateral oblique (MLO) views in Figure 1a. There is a small amount of gas posteriorly in the left cranio-caudal (CC) view in Figure 1b. There was no evidence of micro-calcification or carcinoma. The patient did not complain of pain during compression of her breasts. She had screening mammograms in September 2022 and May 2021 where no abnormalities were noted.

**FIGURE 2:**
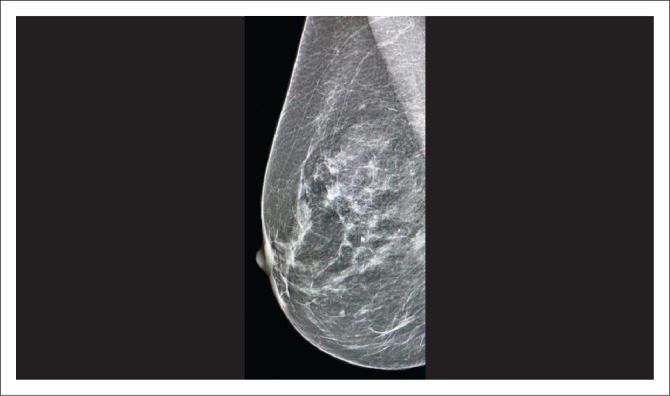
There was no gas visible on the right medio-lateral oblique at the 6-week follow-up mammogram.

In order to establish a reason for the gas, she was questioned about any recent procedures. She stated that she had a bilateral brachioplasty procedure 2 weeks earlier. The procedure was vibration amplification of sound energy at resonance (VASER)-assisted liposuction, coupled with Renuvion skin tightening by J-Plasma for skin retraction. Being asymptomatic, she was managed conservatively, and a 6-week follow-up right MLO mammogram (Figure 2) showed no evidence of the previously visualised gas.

## Discussion

Vibration amplification of sound energy at resonance liposuction^4,8^ and the use of Renuvion (formerly J-Plasma) are a minimally invasive skin rejuvenation and skin firming treatment.^9^ It is commonly paired without liposuction for optimal body contouring results. A surgeon, when treating a combination of extra fat and lax skin, will initially remove the fat via liposuction. A Renuvion cannula is then inserted into the lipo incision to tighten the skin. Renuvion delivers a precise stream of cold plasma energy. The latter is created by energising helium gas with radiofrequency (RF) waves.^10^ It is used as a resurfacing treatment by being applied directly to the skin or subdermally by means of a long, thin tube called a cannula.^10^ The helium cools the skin; thus there is less dermal injury to the superficial skin. Subdermally, the combination of high-energy heat and the plasma’s cold temperature causes collagen to contract as well as tighten the skin.^11^ The device, via tiny incisions, delivers energy to the underside of the skin; this instantly causes firming from the inside-out, with tissue cooling by the helium gas.^12^ The Apyx Medical Renuvion device may be used for patients who do not require excisional surgery for apparent skin laxity.^13,14^

Complications reported in the literature occurred shortly after liposuction and body contouring treatment. Kim et al.^15^ believe that the traumatic subcutaneous emphysema in their patient following suction-assisted liposuction was caused by the entry of air via the skin, which led to the accumulation of air in the fascial planes. Lim et al.^4^ propose that the complications that occurred in their patient were probably because of ultrasound emulsification and liposuction caused by air entrapment, by means of 1-way air entry via skin incisions resulting in a gradual spread of air along the fascial planes. They are of the opinion that the use of J-Plasma, or its combination with liposuction, probably caused the extensive emphysema in their patient. The authors, of a rare complication of surgical emphysema under the right diaphragm in their female patient, suggest this may have accidentally happened because a small calibre cannula is used for tumescent solution infiltration or liposuction;^6^ the muscle and posterior rectus sheath in females is usually thin.

In the presented case, the patient did not experience any pain or discomfort; hence she was unaware of the presence of gas in her breasts when undergoing a screening mammogram 2 weeks after her brachioplasty (Figure 1). As described above, the use of helium is introduced by means of a Renuvion cannula into small skin incisions.^10,11^ The helium ignites the process of skin tightening treatment and can travel along the fascial planes. Breast anatomy includes Camper’s and Scarpa’s facia, which are superficial fascia.^16^ We propose that the gas in our patient tracked from the upper arm small incision sites along Camper’s and Scarpa’s facia to enter the breast tissues. As shown in Figure 2, there was no evidence of gas 6 weeks after the initial mammogram.

## Conclusion

This case underscores the important role of questioning patients in order to consider possible causes of gas in the subcutaneous tissue. A procedural complication, of no clinical concern, was observed on a screening mammogram. There was a collection of gas in the superior and posterior aspects of both breasts. The patient had no symptoms or complaints during the examination. She was unaware of the presence of gas, but on questioning, she reported having undergone liposuction together with the use of Renuvion (J-Plasma) to her upper arms 2 weeks before the mammogram. With the increasing use of this treatment, it is suggested that there may be more cases of surgical emphysema. It is evident that the complication in this case was self-limiting as the gas had resorbed when a 6-week follow-up single-view mammogram was performed.
